# Electro-oculography in bilateral optic neuropathy

**DOI:** 10.1186/s13104-020-05131-0

**Published:** 2020-06-12

**Authors:** Padmini Dahanayake, Tharaka L. Dassanayake, Manoji Pathirage, Saman Senanayake, Mike Sedgwick, Vajira Weerasinghe

**Affiliations:** 1 Department of Physiology, Faculty of Medicine, University of Peradeniya, Peradeniya, 20400 Sri Lanka; 2 Teaching Hospital, Peradeniya, 20400 Sri Lanka; 3 School of Psychology, The University of Newcastle, Callaghan, NSW 2308 Australia; 4 Department of Medicine, Faculty of Medicine, University of Peradeniya, Peradeniya, 20400 Sri Lanka; 5 Eye Unit, National Hospital, Kandy, 20000 Sri Lanka

**Keywords:** LP:DT ratio, Bilateral, Electro-oculography, Optic neuropathy

## Abstract

**Objective:**

Effect of bilateral, optic neuropathy on the function of retinal pigment epithelium has not been investigated extensively to date. This study aimed to determine the effect of bilateral, optic neuropathy on light peak:dark trough ratio, light peak and dark trough values of electro-oculography. Thirty-seven patients with a clinical diagnosis of bilateral optic neuropathy and 40 control subjects were recruited in this observational, cross-sectional study, carried out at the Neurophysiology unit, Teaching Hospital, Peradeniya, Sri Lanka. Pattern reversal visual evoked potentials, pattern electroretinography and electro-oculography were recorded in all of them.

**Results:**

Twenty-four patients (64.9%) had reduced light peak:dark trough ratio values. The median light peak:dark trough ratio ± inter quartile range of the patient group (1.50 ± 0.4) was significantly lower than that of the controls (1.80 ± 0.2), (p < 0.001). Median dark trough value was significantly higher in patients compared to the control value. These changes may be due to higher conductance of ion channels in the retinal pigment epithelium, sub retinal space creation, changes in choroidal circulation or secondary to photoreceptor abnormalities in the macular region. These results indicate that retinal pigment epithelium might be affected in bilateral optic neuropathy.

## Introduction

Acute demyelinating optic neuropathy is a clinical diagnosis. The history points to the aetiology of the optic neuropathy [1]. Pattern reversal visual evoked potentials (PRVEP) is a highly sensitive test of demyelinating optic nerve damage [2]. The retinal pigment epithelium (RPE) is assumed to be unaffected in optic neuropathy, but has not been studied. Its function can be tested by electro-oculography (EOG) which allows an assessment of the standing potential across the RPE [3]. The ratio of the potential between light and dark adaptation, known as the LP:DT ratio (light peak:dark trough ratio), is an essential diagnostic test in retinal disorders [4, 5]. A study done in patients with ischemic optic neuropathy showed a decrease in the EOG potentials presumably due to ischemia in the RPE [6]. The EOG in other forms of optic neuropathy has not been reported.

Optic neuropathy is commonly associated with multiple sclerosis in patients reported in the Western literature and this is mostly unilateral [7]. Compared to the West, the prevalence of bilateral optic neuropathy is higher in Asians, and the conversion of optic neuropathy into multiple sclerosis is less common [8]. The causation of the higher prevalence of bilateral optic neuropathy in Asia is not known although a post-viral pathology has been implicated [9].

Given the scarcity of evidence of the RPE electrophysiology in optic neuropathy, and higher prevalence of bilateral optic neuropathy in Asia, the aim of this study was to evaluate the effect of bilateral, demyelinating optic neuropathy on the RPE as estimated by the LP:DT ratio, amplitudes of light peak (LP) and dark trough (DT) of EOG.

## Main text

### Materials and methods

#### Study setting and participants

This was a cross-sectional study in which we compared a test group of patients with optic neuropathy and a healthy control group. The study was carried out at the Neurophysiology Unit of the Teaching Hospital, Peradeniya in Sri Lanka between February 2018 to October 2019. The study design and protocols complied with the code of ethics of the World Medical Association Declaration of Helsinki [10].

A sample of 36 subjects per each group was required to observe an effect size of 0.7, with a 90% power at α-error probability of 0.05. Thirty seven patients ≥ 18 years of age with a clinical diagnosis of bilateral optic neuropathy referred by consultant ophthalmologists within 21 days of onset of ocular symptoms were recruited. Patients were diagnosed by the sudden onset of visual impairment, positive relative afferent papillary defect (RAPD) and fundoscopic changes. One of the co authors, who is a specialist ophthalmologist (SS) clinically examined and conducted fundoscopic examination of the potential subjects. None of the recruited subjects had previous eye diseases, evidence of neurological diseases or fundoscopic features of other eye pathologies that might confound our results. The control group comprised 40 healthy subjects of age ≥ 18 years with normal or corrected-to-normal vision in neuro-ophthalmologic examination. All subjects had a neuro-ophthalmologic assessment which included; measurements of visual acuity, visual field, colour vision, pupillary reflexes, fundoscopy and ocular motor examination at the referring ophthalmology units. Then visual electrophysiological tests viz. PRVEP by Natus machine (USA), pattern electroretinography (PERG) by Nicolet Viking Quest machine (USA) and EOG were performed in each subject conforming to International society for clinical electrophysiology of vision (ISCEV) guidelines [4, 11, 12].

#### Assessment of electro-oculography

The EOG waveforms were recorded using the Natus EMG/NCV/EP machine (USA). Pupils were not dilated as it was shown that application of pupillary dilatation does not influence the quality or the results of electro-oculograms [13]. The bitemporal method was used to record EOG waveforms. Silver/silver chloride electrodes were used. The active and reference electrodes were attached to the outer canthi of each eye, thus collecting a compound potential difference resulting from both eyes. The ground electrode was placed at Cz. Band pass filters were set to 0.1 Hz and 30 Hz. A Ganzfeld dome provided the stimulation. Two fixating lights were located in the Ganzfeld dome, 15° apart left and right of centre. The patient was kept in stable indoor lighting for at least 30 min before the test. Fixation lights in the Ganzfeld dome were set to alternate at a frequency of once per second, for 10 s out of every minute. The EOG potentials were recorded for once per second for 10 s every minute as the eyes moved to left and right according to the alternating lights in the Ganzfeld dome. Auditory cues were used during the recordings. The procedure of making saccades was practiced with the recording system before dark adaptation, to familiarize the patient with the task and to check on the stability and quality of the recorded saccades. The same procedure was used to test the control subjects. EOG recording began with the beginning of the dark adaptation; the EOG potentials being recorded once a minute for 10 s. The dark phase of the EOG potentials lasted for 15 min. Then the room lights and adapting light of Ganzfeld dome were switched on. The adapting light of Ganzfeld dome was a white light with a luminance of 100 cdm^−2^. The light phase recording lasted for another 15 min [4]. The patient was positioned in the headrest of the Ganzfeld stimulator throughout the procedure, with eyes open to maintain retinal illumination. The EOG amplitudes were measured in microvolts (μV) manually after visual inspection. The effects of overshoot or irregular saccades were avoided.

The average of the EOG amplitudes within each 10 s recording epoch was taken and plotted against time. Then the underlying physiologic curve was drawn using computer-based curve fitting algorithms to derive reliable dark trough (DT) and light peak (LP) amplitudes. Then the LP:DT ratio was calculated by dividing the smoothed light peak by dark trough value for each subject.

#### Data analysis

Continuous outcomes measures, i.e. PRVEP latencies, PRVEP amplitudes, PERG latencies, PERG amplitudes, amplitudes of light peak and dark trough, LP:DT ratios showed skewed distributions, and hence are reported as medians and inter quartile ranges (IQR) and were compared between groups using Mann–Whitney U test. The comparisons were interpreted as significant at a cut-off p value < 0.05. IBM SPSS statistics for windows, version 22.0. was used to analyze the data and Graph Pad Prism version 5.03 was used to create the graphs.

### Results

#### Demographic and clinical characteristics

There were 37 patients with bilateral optic neuropathy (15 (40.5%) males; median age: 47.0 ± IQR 23; range 18–70 years) in the test group and 40 subjects in the control group (34.8% males; median age: 34 ± IQR 18; range 24–60 years). All patients had a clinical history of sudden onset functional visual disturbances, most frequently blurred vision. The median duration to presentation was 10 ± IQR 14 days, with a range of 2–21 days. Fourteen (37.8%) patients had normal fundoscopy and 23 (62.1%) showed optic disc edema. Sixteen had magnetic resonance imaging (MRI) and 4 showed features of multiple sclerosis, 12 had a normal MRI. Twenty three (62.1%) had ocular pain at the onset of the clinical symptoms, but none had pain at the time of the visual electrophysiological examination. RAPD was positive in 24 (64.9%) patients. Median visual acuity by logarithm of the minimal angle of resolution (logMAR) units was + 0.50 ± 0.8 in the right eye and + 0.20 ± 0.5 in the left eye.

#### Pattern reversal visual evoked potentials

Median N75, P100 and N145 latencies in both eyes were significantly prolonged in patients with optic neuropathy when compared with the control group (see Additional file 1: Table S1), signifying demyelinating optic neuropathy.

#### Pattern electroretinography

There was no significant difference in median N35, P50 and N95 latencies and amplitudes of both eyes in the patients compared to the control group (see Additional file 1: Table S2). However, 9 patients in right eye and 11 patients in left eye had delayed P50 latency values, whereas 4 patients in right eye and 5 patients in left eye had absent P50 peak. N95 peak was absent in 7 patients in each eye, whereas 3 patients in right eye and 5 patients in left eye had delayed N95 latencies.

#### Electro-oculography

**Fig. 1:**
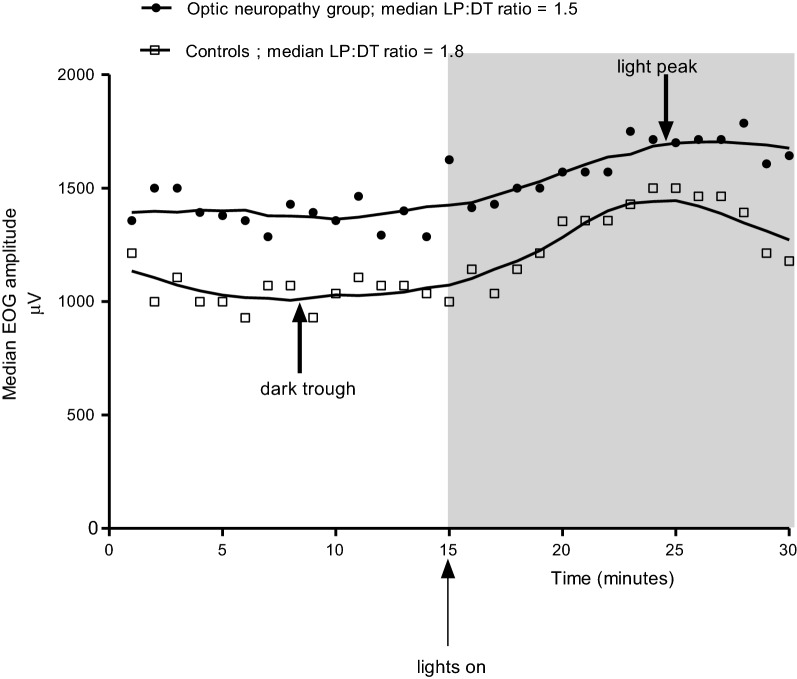
Median EOG waveforms in dark and light phases over 30 min in optic neuropathy group and the controls (LP:DT ratio = light peak:dark trough ratio)

**Fig. 2:**
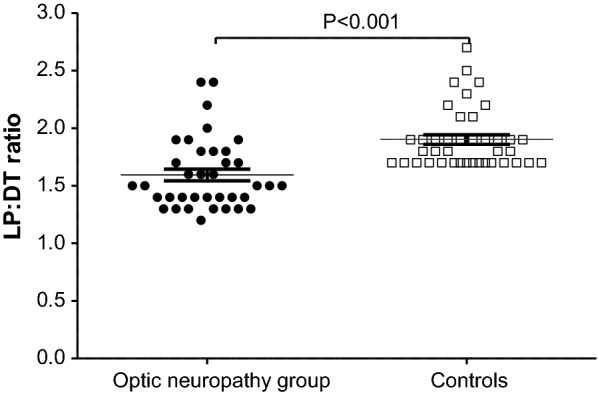
LP:DT ratio values between optic neuropathy group and the controls (LP:DT ratio = light peak:dark trough ratio)

Absolute EOG amplitude values plotted over 30 min, smoothed interpolation curves, and the light peaks and dark troughs are shown in Fig. 1. The median LP:DT ratio ± IQR of the patient group (1.50 ± 0.4) was significantly lower than that of the controls (1.80 ± 0.2) (Fig. 2). Twenty-four (64.9%) patients had an LP:DT ratio below 1.7, which is the ISCEV, cut off lower limit of the normal range and the lowest LP:DT ratio observed in the control group [14]. Median dark trough amplitude of the patient group was significantly larger than that of the controls (Fig. 1, Table 1).

**Table 1:** EOG parameters between optic neuropathy group and controls

EOG parameter	Optic neuropathy group (n = 37) Median ± IQR	Controls (n = 40) Median ± IQR	p value
Light peak (µV)	1860 (984.3)	1700.6 (718.9)	0.16
Dark trough (µV)	1140 (640)	860 (482.5)	*0.001
LP:DT ratio	1.50 (0.4)	1.80 (0.2)	*< 0.001

*IQR* inter quartile range

### Discussion

In the present study, nearly two-thirds (64.9%) of the optic neuropathy patients, had a LP:DT ratio below 1.7, the ISCEV cut-off level. The group difference in the LP:DT ratio seems to be primarily contributed by the greater dark trough amplitudes in the optic neuropathy group.

The RPE forms a barrier between the retina and the systemic circulation, and it performs many vital functions to support the neural retina [15, 16]. As the LP:DT ratio is a manifestation of the corneo-fundal potential generated by the RPE, the results of the present study indicate that significant abnormalities can occur in RPE in a majority of patients with bilateral, demyelinating optic neuropathy. However, it is found that both normal rods and rod photoreceptor-RPE interface are required for a normal EOG [17, 18]. Therefore, abnormality in the EOG is not only diagnostic of abnormality of RPE function but also could be due to dysfunction in rod photoreceptor-RPE interaction as well. The mechanism for the light rise in EOG has been studied since the introduction of the concept of EOG [19]. The current understanding is that a substance is released from the rod outer segment to set off a rise in inositol triphosphate through an apical membrane receptor [20]. This in turn causes a subsequent rise in intracellular free calcium. Then calcium-activated chloride channel will be operated leading to depolarization of the basal membrane of the RPE resulting in initiation of light rise [20–22]. Though many mechanisms have been postulated to explain light rise, still there is no proper mechanism deduced to describe the mechanism of dark trough. Given the role of changes in basolateral chloride conductance regulating the light peak, it is possible to think that the dark trough is generated through a decrease in basolateral chloride conductance [23]. EOG abnormalities observed in our study could be due to a derangement in the chloride conductance which could have occurred as a result of acute episode of optic neuropathy. But the exact mechanism of this ionic abnormality cannot be ascertained.

However, based on the structural and functional abnormalities of optic neuropathy and the unique anatomical arrangement of RPE, alternative mechanisms can potentially contribute for EOG changes that we observed. First, Brudet-Wickel and van Lith reported evidence of partial, temporary disturbance of the choroidal circulation in anterior ischemic optic neuropathy that may cause for the abnormalities of light rise in EOG. Since the choroid is the principal vascular structure nourishing the outer retinal layers and the RPE, disturbance of choroidal circulation could have caused hypoxia of the retinal tissues [6]. This in turn can cause abnormalities in photoreceptors and RPE which is reflected in EOG as a reduction in LP:DT ratio in patients of our study.

Second, disc edema associated with optic neuropathy could lead to accumulation of fluid creating sub retinal space above the RPE [24, 25]. Such sub retinal space could interfere with light reaching the photoreceptors and then alter the surface-recordings of RPE-generated EOG potentials. Third, some patients with optic neuropathy of our study show P50 component of PERG involvement which can be due to photoreceptor damage [26]. This damage in photoreceptor cell layer in turn can cause abnormalities in RPE which closely interact with the photoreceptors [27].

## Conclusions

We conclude that RPE exhibits abnormal function as indexed by reduced LP:DT ratio primarily contributed by the increased dark trough amplitudes in EOG in the majority of patients with bilateral optic neuropathy.

## Limitations

MRI and optical coherence tomography (OCT) facilities were not available readily for every patient due to financial constraints. Also, further studies using a large cohort of patients would add more conclusive evidence.

## Supplementary information

**Additional file 1: Table S1.** Median latencies and amplitudes of PRVEP between optic neuropathy group and the controls. **Table S2.** Median latencies and amplitudes of PERG between optic neuropathy group and the controls.

## Data Availability

The data sets used and/or analysed during the current study are available from the corresponding author on reasonable request.

## Abbreviations

DT: Dark trough
EMG: Electromyography
EOG: Electro-oculography
IQR: Inter quartile range
ISCEV: International society for clinical electrophysiology of vision
LogMAR: Logarithm of the minimal angle of resolution
LP: Light peak
MRI: Magnetic resonance imaging
OCT: Optical coherence tomography
PERG: Pattern electroretinography
PRVEP: Pattern reversal visual evoked potentials
RAPD: Relative afferent pupillary defect
RPE: Retinal pigment epithelium
