# Supplementary material for: Electro-oculography in bilateral optic neuropathy
Source: BMC Res Notes. 2020 Jun 12;13:287. doi: 10.1186/s13104-020-05131-0 (PMC7310542; doi:10.1186/s13104-020-05131-0)
Supplement: Supplementary file 1 — Additional file 1: Table S1. Median latencies and amplitudes of PRVEP between optic neuropathy group and the controls. Table S2. Median latencies and amplitudes of PERG between optic neuropathy group and the controls. [file 13104_2020_5131_MOESM1_ESM.doc]

Table S1: Median latencies and amplitudes of PRVEP between optic neuropathy group and the controls

| Parameter | Left | | | Right | | |
| --- | --- | --- | --- | --- | --- | --- |
|  | Optic neuropathy group *(n=37)*  *Median ± IQR* | Controls *(n=40)*  *Median ± IQR* | P value | Optic neuropathy group *(n=37)*  *Median ± IQR* | Controls *(n=40)*  *Median ± IQR* | P value |
| N75 Latency | 86.4 (23.6) | 76.4(8.5) | *<0.001 | 86.2 (25.9) | 75 (8.0) | *0.001 |
| P100 Latency | 121.6(17.2) | 100.2(11.4) | *<0.001 | 119.7 (12) | 100.5(10.3) | *<0.001 |
| N145 Latency | 156 (24.6) | 140 (9.4) | *<0.001 | 151.9(13.9) | 138.7(12.3) | *<0.001 |
| N75 Amplitude | 1.0(0.50) | 0.9 (0.8) | 0.2 | 1.0(1.0) | 1.0(0.4) | 0.9 |
| P100 Amplitude | 5.2(4.6) | 3.4(4.0) | 0.1 | 5.0(3.6) | 3.5(5.3) | 0.1 |
| N145 Amplitude | 4.2(4.0) | 3.6(3.6) | 0.6 | 5.0(4.0) | 4.0(3.8) | 0.3 |
|  |  |  |  |  |  |  |

IQR: Inter quartile range

Table S2: Median latencies and amplitudes of PERG between optic neuropathy group and the controls

| Parameter | Left | | | Right | | |
| --- | --- | --- | --- | --- | --- | --- |
|  | Optic neuropathy group *(n=37)*  *Median ± IQR* | Controls *(n=40)*  *Median ± IQR* | P value | Optic neuropathy group *(n=37)*  *Median ± IQR* | Controls *(n=40)*  *Median ± IQR* | P value |
| N35 Latency | 39.6(28.6) | 36.7(7.6) | 0.7 | 36.5(16.9) | 37.5(7) | 0.7 |
| P50 Latency | 57.5 (28.3) | 54 (6.9) | 0.1 | 51.7 (9.4) | 56.5(24.3) | 0.1 |
| N95 Latency | 90.0(14.5) | 90.7(5.4) | 0.4 | 92.7 (15.1) | 91.2 (5.4) | 0.7 |
| N35 Amplitude | 0.5 (0.5) | 0.3(0.7) | 0.5 | 0.2(0.4) | 0.2(0.5) | 0.1 |
| P50 Amplitude | 1.4 (1.9) | 1.8(1.4) | 0.4 | 1.2 (1.5) | 1.2 (1.5) | 0.4 |
| N95 Amplitude | 1.2 (2.2) | 1.4 (1.0) | 0.7 | 1.5 (1.3) | 1.4(1.9) | 0.8 |

IQR: Inter quartile range
